# Assessing the equivalency of face-to-face and online simulated patient interviews in an educational intervention

**DOI:** 10.1186/s41077-024-00286-3

**Published:** 2024-04-05

**Authors:** Cheryl Regehr, Arija Birze

**Affiliations:** 1 Factor-Inwentash Faculty of Social Work, University of Toronto, Toronto, Canada; 2 Institute for Better Health, Trillium Health Partners, Mississauga, ON Canada

**Keywords:** Simulation, OSCE, Health professional students, Online

## Abstract

**Background:**

In adapting to COVID-19, many health professional training programs moved abruptly from in-person to online simulated patient interviews for teaching and evaluation without the benefit of evidence regarding the efficacy of this mode of delivery. This paper reports on a multi-methods research project comparing in-person and online simulated patient interviews conducted by allied health professionals as part of an educational intervention offered at a large university teaching hospital.

**Methods:**

Twenty-three participants conducted two 15-min interviews with simulated patients using previously validated scenarios of patients presenting with suicide risk. In order to assess the equivalency of the two modalities, physiological and psychological stress were measured using heart rate variability parameters and the State-Trait Anxiety Inventory respectively, and then were compared across cohorts using *t*-tests. Reflective interviews elicited qualitative impressions of the simulations that were subject to thematic qualitative analysis.

**Results:**

There were no statistical differences in measures of psychological stress or physiological arousal of participant health care professionals who engaged with in-person versus online simulated interviews, suggesting they were equally effective in eliciting reactions commonly found in challenging clinical situations. In reflective interviews, participants commented on the realism of both modalities of simulated patient encounters and that simulated interviews provoked emotional and physiological responses consistent with actual patient encounters.

**Conclusions:**

These findings provide developing evidence that carefully designed online clinical simulations can be a useful tool for the education and assessment of healthcare professionals.

## Introduction

Gaba defined simulation as a technique designed to replace “real life experiences with guided experiences, often immersive in nature, that evoke or replicate substantial aspects of the real world in a fully interactive fashion” [1] (p 126). To this end, simulated patients (alternatively referred to as standardized patients by some researchers and educators—see [2–4]) present realistic patient scenarios, allowing for the demonstration, assessment, and observation of competence in healthcare trainees [5, 6]. Originally designed for medical education [7–9], the simulated patient methodology has subsequently been adapted for education and research purposes for a range of allied health professions [10–13]. Simulated patient methodology has been used to enhance cultural competence among healthcare providers [14, 15]; examine factors affecting clinical reasoning during medical emergencies [16–19]; and investigate decision-making patterns in health professionals such as pharmacists [20, 21], nurses [22, 23], physiotherapists [24], and social workers [25, 26]. It has been suggested that simulated patients can accurately replicate clinical practice in terms of symptom clusters that patients present (referred to as physical fidelity), and can achieve psychological fidelity if the simulated patient is able to accurately portray the emotions of an individual faced with those symptoms, thereby allowing the participant to authentically engage as they would in clinical practice [27].

Physiological stress research has demonstrated that stress responses to simulations can be remarkably similar to responses in actual clinical situations. For instance, Stevens and colleagues found congruence in the neurodynamics of teams (using EEG data) during simulated surgeries and surgeries with live patients [28]. Similarly, Dias and Neto reported equivalent stress responses in real life and simulated emergencies encountered by internal medicine residents, as measured by heart rate variability, salivatory interleukin-1β, and scores on the State-Trait Anxiety Inventory [29]. Importantly, research has determined that high-fidelity simulations evoke physiological and psychological stress responses even in highly trained emergency medical teams [30]. This suggests that simulations that replicate high-risk clinical encounters give us a unique window into clinical performance and decision-making. Among varying types of simulation, some research suggests that simulated patient encounters may be particularly effective in replicating stressful clinical encounters and result in elevated levels of physiological arousal as measured by salivary alpha-amylase activity [17]. For instance, the salivary alpha-amylase response has been found to remain elevated for a more prolonged period following nursing simulations with simulated patients than with high-fidelity mannequins [31].

In general, research on simulated patients has focused on their efficacy in in-person encounters using a variety of parameters such as acquired medical knowledge of trainees [5]; heart rate variability and electrodermal activity [32, 33]; and assessments of the verisimilitude of the scenarios by participants [34, 35]. The onset of COVID-19, however, precluded the use of in-person simulations in teaching and assessment of health professionals and forced programs to rapidly develop online approaches [34, 36–39]. Research conducted in the wake of COVID-19, suggests that online simulated clinical interviews are positively assessed by students and faculty, that they realistically reflect the online counselling environment, and do so in a way that feels “safer” and is less anxiety provoking [34, 36–40]. Notably missing, however, are other measures to assess the ability of online simulated interviews to replicate practice experiences. This research aims to address this gap in evidence and compare the level of physiological and psychological stress experienced by allied health professionals when conducting in-person and online simulated patient interviews assessing suicide risk and the appraisals of participating health professionals on the realism of the simulated experiences.

Previously we have reported on the evaluation of a multi-component educational intervention, aimed at improving professional decision-making among allied health professionals facing situations of risk and uncertainty, one component of which was in-person simulated patient interviews for assessing suicide risk. The simulated patient interviews conducted by participants, and subsequent post-simulation reflective interviews with researchers, provided participants with an opportunity to reflect upon their own decision-making processes [41]. We concluded that the findings suggested that the intervention held promise for refinement and replication. As the COVID-19 pandemic ensued shortly thereafter, and in-person replication was not possible, we designed and implemented an online version of the intervention, including online simulated interviews. This paper reports on multi-methods research aimed at assessing the equivalency between in-person and online simulated patient interviews using physiological and psychological measures and qualitative impressions. Specifically, the research sought to address the following research questions:

1. Do allied health professionals experience the same level of psychological and physiological stress when conducting a simulated patient interview online versus in-person?
2. How do allied health professionals describe their subjective experiences when interviewing simulated patients online versus in-person?

Research ethics board approval was obtained from the University of University of Toronto as well as the Centre for Addiction and Mental Health. All participants provided written informed consent.

## Method

As part of a larger pilot intervention aimed at improving professional decision-making in situations of risk and uncertainty, two simulated patient interviews were conducted by participants. Simulations involved two 15-min interviews using realistic and previously validated client scenarios to assess suicide risk (authors). One client was an adolescent/young adult (Karolina) presenting with a situational crisis; the second was a depressed middle-aged woman (Margaret) who was a victim of intimate partner violence.

Participants were randomly assigned to complete one scenario prior to the educational intervention, and one following the intervention, such that each participant interviewed each simulated patient once. In the in-person iteration, the interviews were conducted face-to-face and in the online iteration, participants interacted remotely with the simulated clients via the Zoom videoconferencing platform. Simulated interviews were video recorded and videos were played back to participants through a guided interview reflection process, during which they examined and explicated their decision-making process, including cognitive, somatic, and affective influences.

Simulated patients were hired from the Standardized Patient Program of the University of Toronto. This program provides trained and experienced simulated patients to all health professions education programs of the University for teaching, research, and clinical examination purposes. The simulated patients in this study were further trained by the researchers and engaged in mock interviews with researchers to facilitate authenticity and accuracy to the standardized scenarios.

### Participants

In both the in-person and online cohorts, allied health professionals were recruited from the Centre for Addiction and Mental Health, a large mental health facility associated with the University, through a flyer sent via the organization’s listserv. The in-person iteration was piloted with the intention of replication but as a result of COVID-19 restrictions, an online version was created. Thus, participation in the in-person versus the online version was not by random assignment. Data for the in-person version was collected in September 2019 for the simulation conducted prior to the onset of the continuing education intervention and in December 2019 for the post-intervention simulation [41]. Data for the online version was collected in September 2022 for the pre-intervention simulation and December 2022 for the post-intervention simulation. All data was collected at the Centre for Addiction and Mental Health.

Prior to both the in-person and online interventions and in conjunction with hospital administrators, all practicing social workers, nurses, and occupational therapists with 3 or more years of clinical experience were extended an invitation to participate in the educational intervention and research study.

### Measures of stress

Acute psychological stress during all simulated patient interviews was assessed using the state form of the State-Trait Anxiety Inventory (STAI) [42]. It consists of 20 statements to which respondents indicate their level of agreement on a 4-point scale regarding how they feel at the given moment. The internal consistency of the STAI-S anxiety scale is high, with alpha coefficients above 0.85. The STAI has been used in a number of studies assessing the ability of simulations to reflect actual clinical practice [29].

Continuous heart rate variability (HRV) is another measure that has been used to assess acute stress in clinical situations [43], stress experienced by participants in clinical simulations [32], and the equivalence of stress response between simulated and real-life clinical encounters [29, 44, 45]. In the present study, HRV was recorded with a FirstBeat BodyGuard 2 HRV monitor, a small and comfortable device affixed to the chest and side with 2 electrode patches [46], that provided data on changes in HRV as an assessment of autonomic nervous system (ANS) activity during simulations. During stress, both the parasympathetic (PNS) and sympathetic (SNS) branches of the ANS are affected; the PNS is suppressed while the SNS increases in activity, thereby increasing HR and decreasing HRV [47–49]. HRV data was analyzed using Kubios Standard 3.3.1. Specifically, the PNS index (PNSi) which serves as a measure of parasympathetic nervous system activity and is expected to decrease during stress and the SNS index (SNSi) which serves as a measure of sympathetic nervous system activity and is expected to increase during stress, are analyzed to provide an overall assessment of stress according to the balance of ANS activity [48].

Continuous HRV recorded during the pre and post-intervention simulated patient interviews, was averaged during 5-min segments (epochs) at six points throughout the session: at baseline; at the beginning, mid-point, and end of the simulated interview; and at 10 and 20 min post-interview. Mean differences in HRV parameters (PNS index and SNS index) between online and in-person participants were compared using independent samples *t*-tests.

Subjective stress was similarly recorded using the STAI at 5 points during the pre and post-intervention simulated interviews: at baseline, at the beginning of the simulated interview, at the end of the simulated interview, 10 min post-interview, 20 min post-interview). Mean differences in STAI scores between online and in-person participants were compared using independent samples *t*-tests. All statistical analyses were conducted using SPSS Statistics v. 28.0.0.1 (14).

### Qualitative analysis

As noted above, following each simulation, participants engaged with researchers in a reflective interview. While a primary purpose was to reflect on decision-making, probes also included asking about physical and emotional responses during the simulation and whether the experience reminded them of any other clinical or personal encounters. These reflective interviews were recorded and transcribed for analysis.

Paying particular attention to participants’ subjective experiences of the simulated interviews, transcripts were then subjected to a thematic analysis approach [50–52] which is “a tool or technique, unbounded by theoretical commitments…that provides accessible and systematic procedures for generating codes and themes from qualitative data” [53] (p 297). It involves six phases: familiarization with the data; coding; searching for themes; reviewing themes; defining and naming themes; and writing up. In this study, the authors (a senior researcher in the area of workplace stress and decision-making, and a post-doctoral fellow working with the senior researcher) familiarized themselves with the transcripts, highlighting comments related to participants’ qualitative assessments of the simulated patients and their impacts. Following independent manual coding, codes were then synthesized into initial themes, which were then reviewed by the other team members, and themes were collaboratively named. In this process, we identified commonalities and differences within cohorts (online versus in-person) and between cohorts. A selection of participant quotes has been included to illustrate themes in the results section [50–52].

Trustworthiness in qualitative research has traditionally focused on verisimilitude or the appearance of truth [54], that is, achieving a sense of resonance or congruence with the audience who may have experienced similar situations [55]. This is similar to the construct of credibility proposed by Lincoln and Guba [56]. A primary method for achieving this is through triangulation [57]. In this study, this was achieved through triangulating qualitative results with quantitative findings and with the research literature. Additional methods for ensuring trustworthiness in this study involve prolonged engagement of the researchers in using simulation methods to examine clinical decision-making; discussing initial themes with participants during the educational intervention; and peer debriefing with other researchers in the field.

## Results

Thirteen individuals voluntarily engaged in the in-person intervention, 11 women and 2 men. The mean age was 38 with an age range of 25–50. Seven participants identified as White and six as members of other racial groups. Participants had worked an average of 10.3 years (range 1.5–23) in the professions of social work (8), nursing (4), and occupational therapy (1). Ten individuals voluntarily engaged in the online intervention, 9 women and 1 man. The mean age was 44 with an age range of 25–59. Six participants identified as White and four as members of other racial groups. Participants had worked an average of 16.4 years (range 1–36) in the professions of social work (7), nursing (2), and occupational therapy (1).

### Stress responses during simulations

**Fig. 1:**
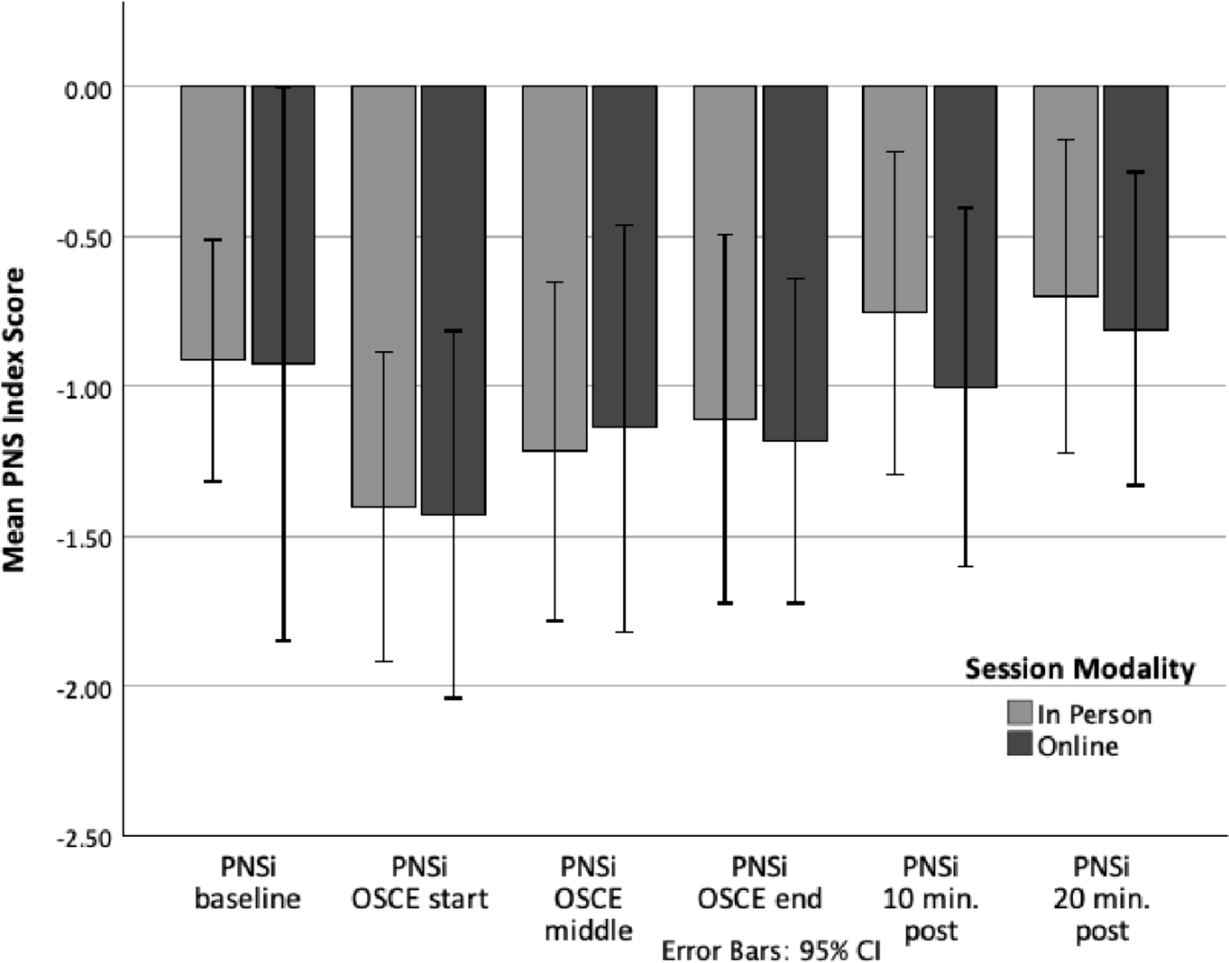
Between-group comparison of changes in Parasympathetic Nervous System Index (PNSi) scores (heart rate variability parameters) during an educational intervention using simulated patient interviews

**Fig. 2:**
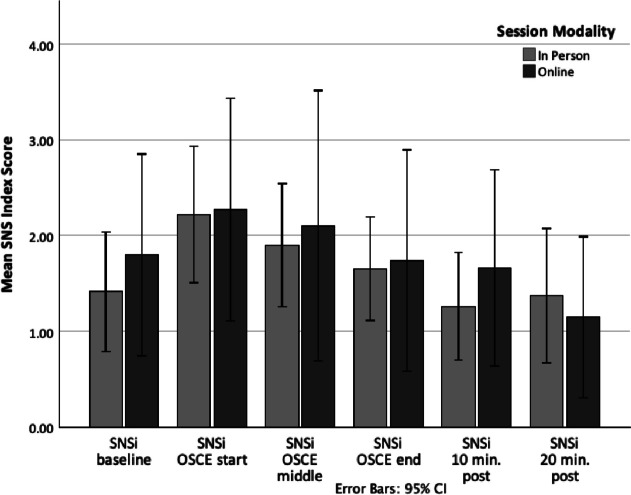
Between-group comparison of changes in Sympathetic Nervous System Index (SNSi) scores (heart rate variability parameters) during an educational intervention using simulated patient interviews

Two forms of stress response were measured in this study, physiological stress (as measured by HRV) and psychological stress (as measured by the STAI). Parasympathetic nervous system tone or the PNSi (a compilation of HRV parameters including mean RR, RMSSD, and SD1(%); [48, 58]) was at its lowest point at the beginning of the simulated interviews and then reached its highest point after the simulations had ended, as is commonly found during a stressful clinical encounter. In an opposite fashion, sympathetic nervous system tone or the SNSi (a compilation of HRV parameters including HR, Stress Index, and SD2(%); [48, 58]) was at its highest point at the beginning of the simulated interviews and then reached its lowest point after the simulations had ended, again, as in commonly found during a stressful clinical encounter. That is, while physiological stress remained relatively heightened throughout the session, by the final assessments, participants had returned to lower than baseline levels of physiological stress. Of note, independent samples *t*-tests revealed no statistical difference in HRV parameters (PNSi and SNSi) at each time point between the two models of delivery, with all mean differences falling within a 95% confidence interval (see Table 1). Additionally, all confidence intervals crossed zero, further supporting no significant difference in HRs between groups and a mean difference of zero as a reasonable population estimate. This suggests that the two delivery methods were equally able to produce physiological arousal (see Figs. 1 and 2; [59]).

**Table 1:** *t*-test results

Name	*t*(df)	Significance	95% CI
HRV PNSi			
T1	*t*(34) = .027	.98	[− .836, .858]
T2	*t*(35) = − .029	.98	[− .781, .759]
T3	*t*(35) = − .272	.79	[− .956, .730]
T4	*t*(35) = .092	.93	[− .804, .880]
T5	*t*(35) = .534	.60	[− .581, .995]
T6	*t*(35) = .207	.84	[− .665, .816]
HRV SNSi			
T1	*t*(34) = − .714	.48	[− 1.487, .714]
T2	*t*(35) = .022	.98	[− 1.201, 1.228]
T3	*t*(18.55) = − .214	.83	[− 1.661, 1.353]
T4	*t*(18.84) = − .079	.94	[− 1.282, 1.189]
T5	*t*(20.92) = − .673	.51	[− 1.491, .762]
T6	*t*(35) = .473	.64	[− .801, 1.287]
STAI			
T1	*t*(22.6) = − .595	.56	[− 8.70, 4.81]
T2	*t*(38) = .147	.88	[− 6.75, 7.80]
T3	*t*(40) = − .382	.70	[− 8.52, 5.81]
T4	*t*(20.6) = − .459	.65	[− 9.90, 6.32]
T5	*t*(40) = .751	.46	[− 3.79, 8.28]

**Fig. 3:**
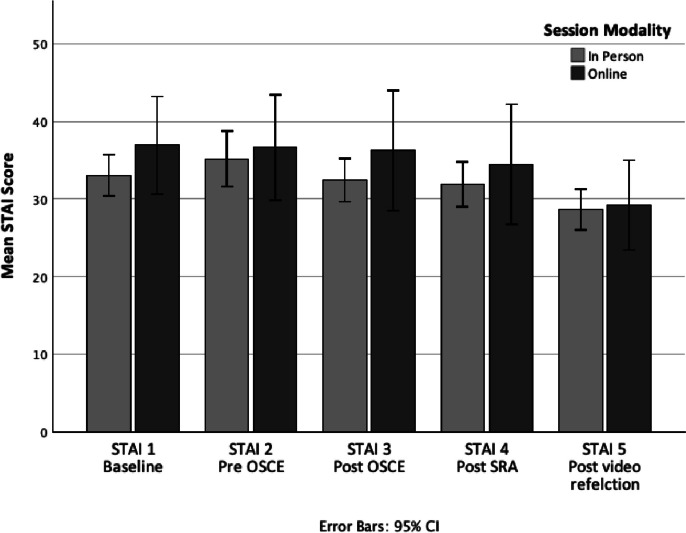
Between-group comparison of changes in subjective stress (STAI) scores during an educational intervention using simulated patient interviews

In a similar manner to HRV, mean STAI scores rose from baseline to a high point at the onset of the simulated interviews, and then diminished during the recovery period. That is, while subjective stress remained heightened and relatively stable throughout the session, by the final assessments, participants had returned to lower than baseline levels of subjective stress. In reflective discussions, participants affirmed that they similarly experienced the highest levels of psychological arousal as they entered real-life high-risk decision-making situations. While mean scores on the STAI were slightly higher for the online group, there are no statistical differences in STAI scores between the two models of delivery, with all mean differences falling within a 95% confidence interval (see Table 1). The observed difference in means related to the influence of one individual reporting higher levels of stress. Additionally, all confidence intervals crossed 0, further supporting no significant difference in STAI scores between groups and a mean difference of 0 as a reasonable population estimate. This suggests the two delivery methods were equally able to produce psychological arousal or stress (see Fig. 3; [59]).

### Qualitative findings

The thematic analysis of post-simulation reflections across cohorts generated a series of themes suggesting participants experienced robust clinical and psychological realism regardless of their cohort. In line with the quantitative findings reported above, individuals who engaged with online simulated patients indicated that the “simulation was very realistic” (OL-109). One participant explains that almost all of her client work is in-person, and yet “I felt like she was right here in front of me, it felt like we were in person.” (OL-101) Identified themes were: comparisons with previous clinical encounters; emotional engagement; and physiological arousal.

#### Comparisons with previous clinical encounters

First, participants consistently used the word “client” when describing their simulations and made comparisons to real-life client encounters. This suggests a degree of commitment and buy-in to the simulations even after they were completed. For example, a participant in the in-person cohort says “I feel empathy, sadness towards the client. She could be any of the clients that I used to work with.” (IP-008), while a participant in the online cohort recalls “I actually had a very similar client back when I worked within the community health centers and I was working with kids. The [current] client is actually a little older. I worked with a 14-year-old who was the same.” (OL-105).

#### Emotional engagement

Second, participants suggest similar levels of complexity while trying to emotionally engage and connect with the simulated patients, whether encounters occurred online or in person. For example, a participant in the in-person cohort recalls the encounter “sometimes I did feel like I was clicking with her, and we were getting somewhere, but then at other times, I felt that I was walking on eggshells.” (IP-002). Participants in the online cohort recall “I had a sense of connection, like I had a sense and that made me feel good. It made me feel like I was connecting with this client, that I was building rapport with this client…and I felt a little afraid of what would happen if I let her go home.” (OL-102) and “I noticed she connected with me. When she made eye contact the first time, I noticed that, and I thought, okay, I’m on the right track [but] I was aware that I wasn’t going to get the whole assessment completed from probably the beginning…” (OL-104).

#### Physiological arousal

Third, participants in both cohorts recognized and articulated similar physiological sensations and responses to the encounters such as racing heart rate and stomach flips as a result of the interactions. For instance, a participant in the in-person cohort describes “I felt certainly a surge of my heart rate, of my brain going a bit blank for a minute, so signs of feeling a bit anxious.” (IP-004). Similarly, in the online cohort, another describes “I wouldn’t say I’m sweating but I can definitely say my heart’s racing.” (OL-105) while another recalls “When she mentioned her mom…it was just a little moment. Like I could feel my stomach.” (OL-106).

## Discussion

Simulated patient encounters through working with simulated patient interviews have become essential tools in clinical education [5–8] and clinical research [16–19]. From a teaching perspective, they are used to teach clinical skills, cultural competence, interprofessional collaboration, and assess clinical competencies. From a research perspective, simulated patient interviews can help elucidate aspects of clinical practice and professional decision-making that are difficult to ascertain due to logistical and ethical constraints of real-life practice [19]. Critical to the effective use in both teaching and research, however, is the degree to which simulations truly reflect actual practice [60, 61]. Most research to date has focussed on the efficacy of simulated patient interviews in face-to-face encounters. As COVID-19 required the rapid transition of simulated interviews to online formats [34, 36–39], a need arose to determine their ability to replicate practice in the virtual realm.

We have previously demonstrated the effectiveness of simulated patient interviews in creating a stressful decision-making situation that might closely reflect a real-life practice encounter [62]. This study sought to determine whether an online synchronous interview with a simulated patient was as effective in replicating a clinical encounter as an in-person simulation. In doing so, we considered multidimensional aspects including assessments of the verisimilitude and clinical realism of the scenarios by participants [34, 35, 63], physiological responses during simulations [30, 32]; and the ability to elicit emotional responses and develop a sense of connection with the simulated patient [64].

To this end, physiological stress as measured by HRV parameters, and psychological stress as measured by the STAI, demonstrated that both in-person and online interventions were effective in eliciting symptoms of stress commonly found in stressful work situations. This is consistent with previous laboratory and simulation research [32, 65, 66]. In addition, the comments of participants supported the realism of an online simulation, a finding that replicates that of others in a variety of clinical fields [40, 67, 68]. Furthermore, comparisons across and within cohorts suggest a number of thematic similarities in both the clinical and psychological verisimilitude experienced by participants. Both cohorts discussed interactions with simulated patients using language that suggests engagement and presence [69] through cognitive and emotional connections and scenario realism that was well aligned with their real-life clinical experiences.

While technology-assisted models of education and clinical practice were on the rise prior to COVID-19, their use accelerated dramatically during the pandemic and has remained higher as the crisis wanes. Not only do online options increase the accessibility of education and clinical services for those who are challenged by mobility, transportation, time, and location, but there is emerging evidence that online simulated patient interviews realistically reflect the online counselling environment, doing so in a way that feels “safer” [34, 36–40], perhaps thereby enhancing learning. In a world where virtual modes of clinical teaching, research, and care provision are key innovations and are no doubt here to stay, ensuring the efficacy of online simulations is critical.

### Limitations

This study demonstrates the challenges and limitations of a real-world intervention. First, the study began as a small in-person pilot with the intention that subsequent iterations would result in larger numbers of participants to test the model. A global pandemic thwarted these intentions and resulted in a reworking of the model as an online intervention. Nevertheless, the aftermath of COVID-19 in the hospital sector resulted in continuing workforce challenges and we were only able to recruit another small cohort for the second iteration. Thus, the result is a small pilot study with only 13 participants in the in-person and 10 participants in the online, conducted in one organization. While we found no significant differences between groups, the small sample size may have resulted in a failure to detect differences. Nevertheless, that mean differences fell within 95% confidence intervals that crossed zero is heartening. Other limitations include: random assignment into the two modes of delivery was not possible given the exigencies that existed; the overrepresentation of women in the study groups; the absence of measures to reduce confounds that may have influenced stress levels measured by heart rate variability and the STAI; and that stress responses were limited to two measures. The generalizability of findings is therefore limited.

## Conclusions

Simulated patient interviews have become a critical component of research and education in the health sciences. As universities across the globe were faced with the challenges presented by the COVID-19 pandemic, this tool for teaching, research, and evaluation of competency was quickly transferred from in-person approaches, which are well supported by research, to online despite the absence of research to support this mode of delivery. This represents a significant gap in the literature. In an effort to assess the equivalency of online and in-person simulated patient interviews, this paper compares online simulated patient interviews with earlier piloted in-person versions. Results are highly promising and demonstrate that a carefully constructed online simulated interview can result in psychological and physiological arousal that is equivalent to an in-person iteration. This is very encouraging, as online delivery creates the potential for the use of simulated patient interviews in a broader range of contexts, supporting online education and research more generally, and making simulation more accessible to community-bound learners and those with mobility and transportation challenges. Further research in this area with larger sample sizes will assist with determining the generalizability of our findings in other contexts.

## Data Availability

The quantitative datasets collected and analyzed during the current study are available from the corresponding author on reasonable request.

## Abbreviations

OSCE: Objective Structured Clinical Examination
EEG: Electroencephalogram
STAI: State-Trait Anxiety Inventory
HRV: Heart rate variability
ANS: Autonomic nervous system
PNS: Parasympathetic nervous system
SNS: Sympathetic nervous system
PNSi: Parasympathetic Nervous System Index (compilation of HRV parameters including Mean RR, RMSSD, and SD1(%))
SNSi: Sympathetic Nervous System Index (compilation of HRV parameters including HR, Stress Index, and SD2(%))
Mean RR: Mean time interval between successive R-waves of the heartbeat
RMSSD: Root mean square of successive differences between RR intervals
SD1(%): Poincaré plot standard deviation perpendicular to the line of identity
HR: Heart rate
SD2(%): Poincaré plot standard deviation along the line of identity
OL: Online
IP: In-person
